# A study about the relationship between sociodemographic characteristics, spirituality, and mental health among emerging adults in metro Manila: findings on higher risk for mental health conditions among LGBTQ+ emerging adults

**DOI:** 10.3389/fsoc.2024.1452701

**Published:** 2024-11-26

**Authors:** Penelope M. Wong, Jasmine Eve C. Ong, Jasper S. Chua, Annika Shanice C. See, Rowalt Alibudbud

**Affiliations:** Department of Sociology and Behavioral Sciences, De La Salle University, Manila, Philippines

**Keywords:** sociodemographic characteristics, LGBTQ+, spirituality, religion, mental health, emerging adults, metro Manila, Philippines

## Abstract

**Introduction:**

Mental health disorders are the third most common disorder in the Philippines, showing a need for further studies in this field among the Filipino population. Several studies have shown that sociodemographic characteristics and spirituality could influence the mental health of individuals, although there are sparse studies in the Philippines.

**Objectives:**

The present study explored the relationship between sociodemographic characteristics and spirituality to depression, anxiety, and stress among emerging adults in Metro Manila.

**Methods:**

The study gathered data using a self-administered sociodemographic characteristics questionnaire, the Core Dimensional Spirituality Questionnaire (CDSQ), and the Depression, Anxiety, Stress Scale (DASS 21). The data collected was then analyzed through linear regression analysis.

**Results:**

More than half of the participants had significant scores for anxiety, while two out of five participants had significant scores for depression. Almost one out of five participants had significant scores for stress. Depression scores are positively associated with being LGBTQ+ and belief in God, while they are negatively associated with feelings of security. Similarly, anxiety scores are positively associated with being LGBTQ+. Likewise, stress scores are positively associated with being LGBTQ+ and age, while it is negatively associated with feelings of security.

**Conclusion:**

The findings suggest that LGBTQ+ emerging adults and those with older age may need additional focus in mental health programs. Likewise, mental health programs may also enhance their activities to increase an individual’s security.

## 1 Introduction

Mental health remains one of the socially relevant issues faced by the Philippines, as the third most common disability in the Philippines is mental disorders. Around 6 million Filipinos are living with anxiety and depression. This is the highest rate in the Western Pacific region (Maravilla and Tan, 2021). Although the government has begun to address mental health issues, a mere 5% of the healthcare expenditure is historically utilized for mental health services (Maravilla and Tan, 2021). Subsequently, effort must be made to respond to these conditions.

The COVID-19 pandemic has impacted the mental well-being of Filipino youth—owing to one of the strictest and longest lockdowns compounded with poverty (Alibudbud, 2024). The country’s mental health conditions, already worsened by the pandemic, may have received little priority. High costs, limited primary care services, and poor record-keeping have exacerbated the situation (Cureg et al., 2023). Social stigma also remains prevalent, manifesting as family rejection, and unrealistic and oversimplified perspectives on mental illness (Maravilla and Tan, 2021).

Sociodemographic characteristics and spirituality influence mental health (Benatov et al., 2022; Leung and Mu, 2021). For example, individuals younger than 35 years of age have worse mental health during the COVID-19 pandemic (Pieh et al., 2020). Likewise, symptoms of anxiety and depression increase from late childhood through early adulthood for many individuals (Gruber et al., 2020; Hawes et al., 2021). Furthermore, sociodemographic characteristics influence mental health. Particularly, gender, which develops from biology, where individuals are socialized differently based on their sex and this thus allows them to become gendered beings that can experience patriarchy and sexism—which can lead to poor health outcomes (Heise et al., 2019). Income is also a factor, where experiences of poverty and stigma lead to diminished well-being (Inglis et al., 2022) and lower income individuals are three times likely to experience depression and anxiety (Ridley et al., 2020). Lastly, religious affiliation (Vaingankar et al., 2021) and positive religious coping lead to improved mental health outcomes (Thomas and Barbato, 2020).

Another possible factor of mental health among emerging adults within Metro Manila is spirituality. Spirituality is an inherently inner and personal relationship that concerns one’s purpose, truth, and values (Kao et al., 2020). To the authors’ knowledge, most existing studies come from foreign nations or focus on older demographics (Thomas and Barbato, 2020; Vaingankar et al., 2021). Thus, this study explored the relationship between spirituality, sociodemographic factors, and mental health among emerging adults in Metro Manila.

### 1.1 Sociodemographic characteristics and mental Health

Age, sex, being LGBTQ+, income, and religion have been shown to impact mental health (Ganson et al., 2021; Heise et al., 2019; Pieh et al., 2020; Tan and Saw, 2022; Vaingankar et al., 2021). Studies have shown that individuals younger than 35 showed worse mental health than older individuals (Benatov et al., 2022; Pieh et al., 2020; Solomou and Constantinidou, 2020). An exacerbating factor is loneliness, a factor greatly affecting the mental health of emerging adults (Lee et al., 2020). Essentially, emerging adults, due to higher risks of loneliness, depression, and anxiety, may have worse mental health than younger or older people.

Sex, referring to a person’s biology, influences their culturally defined role of gender as well since sex determines how individuals undergo gender socialization and ultimately, become gendered beings—particularly, being female entails experiencing sexism and patriarchy, which may lead to poor mental health (Heise et al., 2019).

Females generally have worse mental health outcomes than males due to prevailing discrimination and gender inequality, which implies that gender shapes mental health (Hasin et al., 2018; Hawes et al., 2021; Heise et al., 2019; Lee et al., 2020; Pieh et al., 2020; Solomou and Constantinidou, 2020). For example, females in service sectors are exposed to different kinds of stressors than males in labor sectors, which may affect their mental health differently— sexism, the patriarchy, gender inequality, and restrictive gender norms favor males over females restrict them, influencing their well-being (Heise et al., 2019). Furthermore, males show their negative mental health effects through externalized means, such as substance abuse and self-neglect, while females may manifest their stresses internally through stress, depression, and anxiety (Harder and Sumerau, 2018).

Compared to cisgender and heterosexual individuals, LGBTQ+ people can have higher levels of anxiety and depression (Hunter et al., 2021; Tan and Saw, 2022). LGBTQ+ individuals refer to queer and questioning persons, as well as sexual and gender diverse-identified individuals (Soled et al., 2022). It is posited that LGBTQ+ individuals’ mental health is impacted by systemic discrimination (Valdiserri et al., 2019), familial rejection (Kibrik et al., 2018), and social inequalities, such as minority stigma (Akré et al., 2021). Minority stigma can be classified as either distal (i.e., discrimination) or proximal stressors (i.e., internalized homophobia) (Meyer, 2003), and it describes the unique stressors which are experienced by gender and sexual minorities. Overall, these dimensions contribute to the disparities observed between cisgender and LGBTQ+ individuals.

People with lower incomes are also more likely to experience poorer mental health (Ganson et al., 2021; Inglis et al., 2022; Ridley et al., 2020). Wealth may buffer against economic stressors, and as a result, young adults from wealthier households report less psychological distress (Lê-Scherban et al., 2016). Previous studies suggest that poverty and mental illness have a relationship (Inglis et al., 2022; Ljungqvist et al., 2015).

Religion was also associated with improved mental health outcomes and spirituality than those without religious affiliations (Vaingankar et al., 2021). It is associated with increased mental well-being, general coping, and emotional support. Moreover, positive religious coping lessens depressive symptoms (Thomas and Barbato, 2020). Individuals from a diverse set of faiths utilize religious coping by strengthening their relationship with God, resulting in increased mental well-being (Jeppsen et al., 2022).

### 1.2 Spirituality and mental health

Spirituality is an individual’s inner beliefs of meaning, truth, and values (Charzyńska and Heszen-Celińska, 2019; Kao et al., 2020). Nonetheless, religious activities such as religious participation, attendance, and involvement do not necessarily translate into spirituality (Kao et al., 2020). The historical schism between religion and psychiatric practices existed, wherein the inclusion of both fields was deemed unconventional. Although mental health professionals have a favorable view of the spiritual dimensions of therapy, spirituality remains an underutilized tool in managing mental health due to a lack of training and the ethical constraints of coercing the patient (Charzyńska and Heszen-Celińska, 2019; Rosmarin et al., 2013). Thus, this could explain the scarce studies regarding spirituality’s influence on mental health.

Positive spirituality could increase the quality of life and mental health (Piccinini et al., 2021), mitigate mental disorders, and encourage positive psychosocial traits (Sharma et al., 2017). Positive spirituality also leads to better mental health outcomes among older individuals (60 and above), while negative spirituality worsens outcomes (Ede et al., 2021; Palaniswamy and Ponnuswami, 2012). Although a study showed that the spirituality of high school students in Hong Kong does not impact their psychological distress, the same cannot be said for participants from Mainland China (Leung and Mu, 2021). Thus, it is reasonable to assume that owing to geographical location and cultural differences, spirituality’s effect on emerging adults’ mental health could produce varying results.

From the earlier discussions, the following hypotheses are made:

*H1*: The researchers expect older age to be significantly correlated to higher risk to mental health conditions.

*H2*: The researchers expect being female to be significantly correlated to higher risk to mental health conditions.

*H3*: The researchers expect being LGBTQ+ to be significantly correlated to higher risk to mental health conditions.

*H4*: The researchers expect lower income to be significantly correlated to higher risk to mental health conditions.

*H5*: The researchers expect not being Catholic to be significantly correlated to higher risk to mental health conditions.

*H6*: The researchers expect negative spirituality to be significantly correlated to higher risk to mental health conditions.

### 1.3 Objectives and significance

This study explores the association between age, sex assigned at birth, being LGBTQ+, income, religion, and spirituality and its relationship to depression, anxiety, and stress among emerging adults. Its findings may offer valuable insights into the mental health conditions of young adults and how these conditions may be alleviated.

## 2 Methodology

This quantitative cross-sectional research utilized a survey design containing a sociodemographic characteristics questionnaire, the Core Dimension Spirituality Questionnaire (CDSQ), and the Depression, Anxiety, and Stress Scale 21 (DASS-21). Its participants were emerging Filipino adults aged 18 to 29 residing in Metro Manila. The data gathered were analyzed through linear regression.

Before any study procedure, approval was secured. Likewise, informed consent was secured. A list of mental health providers was provided in the survey forms to respond to mental health concerns that may arise while answering them (Mental Health Facilities, 2022; Mental Health PH, 2016). Survey respondents were referred to with coded numbers instead of identifying data. Furthermore, once the study period elapsed, the data stored and collected were deleted.

### 2.1 Population and sampling

The study included emerging adults between the ages of 18 and 29 who are Filipino citizens residing in Metro Manila. The sample size was computed using the statistical power analysis program, G*Power 3 (Faul et al., 2007). The study’s sample size was based on 10 predictors, an effect size of 0.15, an alpha error probability of 0.05, and a power of 0.8. As Cohen (1988) and Lakens (2013) recommended, these parameters are generally acceptable, falling within the standard range for minimum power and alpha error probability, set at 0.80 and 0.05, respectively. Using these parameters, the computed target sample size is 118 participants. Additionally, this study utilized snowball sampling to recruit participants through online sharing, using messaging and social media applications to disseminate the survey. A total of 127 participants were recruited for this study.

### 2.2 Instrumentation

This study utilized a self-administered online survey. Once the participants signed their consent forms, they were directed to the research questionnaire containing various sections. The first section of the survey collected data regarding their sociodemographic characteristics. In the survey, the participants’ age was inputted as a numerical value between 18 and 29. Following this, participants were asked to input their sex assigned at birth as either male, female, or intersex. Afterward, they indicated whether they identified as LGBTQ+ by selecting “Yes” or “No.” Next, the participants inputted their income by selecting one of the income classifications based on the Philippine Institute for Developmental Studies (Ta-asan, 2022). These classifications consist of Rich (at least ₱219,140/month), High income (between ₱131,484 and ₱219,140/month), Upper middle income (between ₱76,699 and ₱131,484/month), Middle class (between ₱43,828 and ₱76,669/month), Lower middle class (between ₱21,194 and ₱43,828/month), Low income (between ₱9,520 and ₱21,194/month), and Poor (Less than ₱10,957/month). Finally, the survey inquired about the participants’ religion by selecting either Catholic or Non-catholic.

The second section of the questionnaire focused on spirituality. The survey utilized the CDSQ, developed by Hardt et al. (2012), to assess the participants’ spirituality. The questionnaire had 20 items, and each was scored either 0 (Did not apply to me at all) or 4 (Applied to me very much or most of the time). Sample items include “I feel the love of God.” All of these focus on either belief in god, search for meaning, mindfulness, or feeling of security (Hardt et al., 2012). Among Filipinos, the CDSQ has a Cronbach alpha of 0.883, displaying consistent validity and reliability in research (Alibudbud, 2023b). For the present study, the Cronbach alpha of CDSQ is 0.933, indicating good reliability.

The third section of the survey assessed the risk of depression, anxiety, and stress among the participants by utilizing the DASS-21, a shortened version of the DASS-42, developed by Lovibond and Lovibond (1995). The DASS-21 consisted of 21 items, each corresponding to either depression, anxiety, or stress. Each item was scored on a 4-point Likert scale ranging from 0 to 3, with 0 referring to “Did not apply to me at all,” while 3 referring to “Applied to me very much, or most of the time.” Sample items include “I found it hard to wind down.” The average scores for depression, anxiety, and stress for each participant were calculated by summing their scores on the subscales. These scores were used to categorize participants based on their risk levels for significant depression, anxiety, and stress by establishing cut-off scores of 10, 8, and 15, respectively, as previously employed in studies with Filipinos (Alibudbud, 2023b). The scale suits clinical and non-clinical settings for mental health screening and monitoring. Notably, the DASS-21 has displayed strong reliability among Filipinos, with Cronbach’s alpha values of 0.899 for stress, 0.861 for anxiety, and 0.863 for depression (Alibudbud, 2023b). For this study, the Cronbach’s alpha of DASS-21 was 0.879 for stress, 0.832 for anxiety, and 0.910 for depression, indicating good reliability.

### 2.3 Data collection

The research information and consent form, containing rights to privacy and confidentiality, were provided at the beginning of the survey. Because some participants may have been uncomfortable with the questionnaire, a list of mental health providers was included in the survey. After the participants gave their informed consent, they completed the sociodemographic characteristics questionnaire, the CDSQ, and the DASS-21. Data collection lasted for a month.

### 2.4 Data analysis

Categorical data was summarized using frequencies and percentages, while continuous data was summarized using means and standard deviations. Afterward, the variables, including the DASS-21 and MSPSS subscales scores, were centralized to reduce errors in statistical inferences (Kraemer and Blasey, 2004). Then, three linear regression models and bootstrapping analysis based on 1,000 bootstrap samples following the method performed by Walters and Campbell (2004), were utilized to determine the factors related to anxiety, depression, and stress. The dependent variables of the linear regression models were the DASS-21 sub-scores for anxiety, depression, and stress of the participants. Conversely, the predictors of the regressions were the participants’ sociodemographic characteristics and the different dimensions of spirituality. The Beta coefficient and standard error were noted to determine the factors influencing anxiety, depression, and stress among the participants. A *p*-value of <0.05 was considered significant, and all statistical analyses were performed using the Statistical Package for the Social Sciences (SPSS) version 25.

## 3 Results

### 3.1 Sociodemographic characteristics of the participants

Table 1 shows that the average age of the participants is 18.874 (SD = 1.512). Majority of them were females, while more than a quarter of them (*n* = 45; 27.6%) identified as LGBTQ+ people. Most participants were also from middle-income or higher-income households, and all were Catholics.

**Table 1:** Descriptive statistics of sociodemographic characteristics, spirituality, and mental health of the participants (*n* = 127).

	Mean	SD	Frequency	Percentage
Sociodemographic characteristics				
Age	18.874	1.512		
Sex				
Male			46	36.2%
Female			81	63.8%
Being LGBTQ+			35	27.6%
Religion				
Catholic			127	100.0%
Non-Catholic			0	0%
Income level				
Poor			1	0.8%
Low income			1	0.8%
Lower middle income			9	7.1%
Middle class			30	23.6%
Upper middle income			44	34.6%
High income			20	15.7%
Rich			22	17.3%
Spirituality scores				
Belief in god	12.449	6.468		
Search for meaning	14.551	4.289		
Mindfulness	16.717	3.070		
Feelings of security	11.504	4.710		
Average scores for mental health				
Depression score	9.142	5.536		
Anxiety score	8.921	4.828		
Stress score	9.803	4.951		

### 3.2 Spirituality

Concerning spirituality, the participants scored highest for mindfulness (*M* = 16.717; SD =3.070), followed by search for meaning (*M* = 14.551; SD = 4.289), belief in God (*M* = 12.449; SD =6.468), and feelings of security (*M* = 11.504; SD =4.710).

### 3.3 Mental Health

For mental health, they scored highest for stress (*M* = 9.803; SD =4.951), followed by depression (*M* = 9.142; SD =5.536) and anxiety (*M* = 8.921; SD =4.828). Table 2 shows the number of participants who significantly scored in the DASS-21. Two out of five participants had significant scores for depression (cut-off score = 10), while more than half of the participants had significant scores for anxiety (cut-off score = 8). Likewise, almost one out of five participants had significant scores for stress (cut-off score = 15).

**Table 2:** Significant scores for depression, anxiety, and stress.

	Frequency	Percentage
Depression	54	42.5%
Anxiety	69	54.3%
Stress	24	18.9%

### 3.4 Model summaries

Table 3 presents the summaries of the regression models used to analyze the relationship between sociodemographic characteristics, spirituality, and mental health among the participants. The adjusted *R*-squares of these models range from 0.079 to 0.187, indicating that 7.9 to 18.7% of the variance in depression, anxiety, and stress scores can be attributed to the predictors. Additionally, the variables in these models collectively show significant predictive power for depression, anxiety, and stress among the participants (*p* < 0.05).

**Table 3:** Model summaries of the regression models.

Model	*R*	*R* square	Adjusted *R* square	Total DF	*F*	*p*
Depression	0.489*	0.239	0.187	126	4.628	<0.001
Anxiety	0.371*	0.137	0.079	126	2.347	0.022
Stress	0.449*	0.201	0.147	126	3.719	0.001

* *p* = <0.05.

### 3.5 Relationship between sociodemographic characteristics and mental Health

Table 4 shows the results of the regression models in determining the relationship between sociodemographic characteristics, spirituality, and mental health among the participants. Depression scores are positively associated with being LGBTQ+ (*B* = 3.947, *p* = 0.005). Similarly, anxiety scores are positively associated with being LGBTQ+ (*B* = 2.377, *p* = 0.034). Likewise, stress scores are positively associated with being LGBTQ+ (*B* = 2.670*, p* = 0.020) and age (*B* = 0.563, *p* = 0.034). Conversely, there was no significant association between sex, income, and mental health scores among the participants. These findings suggest that being LGBTQ+ is a risk factor for depression, anxiety, and stress among the participants. Older age is a risk factor for stress as well.

**Table 4:** Regression analysis of depression, anxiety, stress, sociodemographic characteristics, and spirituality of the participants.

	Depression			Anxiety			Stress		
	*B*	Std. Error	*p*	B	Std. Error	*p*	B	Std. Error	*p*
Sociodemographic characteristics									
Age	0.445	0.313	0.144	0.284	0.288	0.310	0.563*	0.268	0.034
Sex									
Male	Referent								
Female	0.470	1.044	0.649	1.280	1.005	0.196	1.352	0.961	0.171
Being LGBTQ+	3.947*	1.251	0.005	2.377*	1.081	0.034	2.670*	1.100	0.020
Income	−0.025	0.372	0.949	0.152	0.334	0.660	0.105	0.335	0.778
Spirituality									
Belief in god	0.037*	0.094	0.680	0.016	0.086	0.861	0.062	0.084	0.469
Search for meaning	0.071	0.161	0.669	0.206	0.132	0.112	0.101	0.144	0.482
Mindfulness	0.079	0.167	0.646	−0.103	0.138	0.440	0.096	0.172	0.575
Feelings of security	−0.439*	0.144	0.005	−0.225	0.135	0.102	−0.404*	0.124	0.001

* *p* = <0.05.

### 3.6 Relationship between spirituality and mental health

Table 4 shows the results of the regression models in determining the relationship between sociodemographic characteristics, spirituality, and mental health among the participants. Depression scores are positively associated with belief in God (*B* = 0.037, *p* = 0.680), while they are negatively associated with feelings of security (*B* = −0.439, *p* = 0.005). Stress scores are negatively associated with feelings of security (*B* = −0.404, *p* = 0.001). Conversely, there was no significant association between search for meaning, mindfulness, and mental health scores among the participants. These findings suggest that higher belief in God is a risk factor for depression, and higher feelings of security are a protective factor against depression and stress among the participants.

## 4 Discussion

The results showed that being LGBTQ+ could be a risk factor for depression, anxiety, and stress. These results are in line with previous studies, which found that LGBTQ+ persons had greater levels of anxiety and depression than their peers (Hunter et al., 2021; Tan and Saw, 2022). Depression, anxiety, and stress may have been aggravated by the negative effects of social stigma and discrimination caused by being LGBTQ+ and low social support (Alibudbud, 2023a; Alibudbud, 2023b; Akré et al., 2021; Cleofas and Alibudbud, 2023; Valdiserri et al., 2019). Beyond the stigma from society-at-large, familial response may play a role in the mental health of LGBTQ+ persons. A study states that family acceptance plays a protective role, acting as a buffer against depression, and thus manages to predict greater mental health among LGBTQ+ young adults (Ryan et al., 2010).

The study also observed that a higher belief in God could be a risk factor for depression. Although religious beliefs may act as a source of hope and meaning, allowing people to cope better, they can also heighten a person’s sense of guilt as feelings of failure to live up to the expectations of their faith emerge (Bonelli et al., 2012). Religious affiliations may also have varying effects on individuals. For some, it may sever them from their communities, while for others it may connect them to social support. Moreover, negative religious coping, which is behavior that includes feelings of abandonment by God and spiritual struggle, tension, and doubt, may also lead to poor mental health (Lawrence et al., 2016).

During the COVID-19 pandemic in the Philippines, factors such as financial problems, fear of infection, and exposure to negative news, compounded with the use of negative religious coping, resulted in a greater risk of depression (Cacho and del Castillo, 2022). Conversely, with the Philippines being a predominantly Catholic nation (Libiran et al., 2024), religious beliefs may be a tool to dismiss mental health conditions. Examples of such notions are the belief that God oversees a person’s fate and that mental illness can be ‘cured’ through faith (Martinez et al., 2020). Despite spirituality’s protective effects on mental health, this is not often the case for LGBTQ+ persons of that faith. For example, the church’s stigmatization and condemnation of diverse sexualities and identities, leads to humiliation and shame among LGBTQ+ people. Thus, despite their solace through faith, the church’s teachings can have negative impacts, including low social support (Libiran et al., 2024). Overall, individuals experience and internalize their beliefs in God differently, and thus, have varying effects on their mental health outcomes.

The study found that older age may be a stress risk factor. Adverse social conditions among older adults, such as mortality, morbidity, poorly fitting housing, and low income, could result in greater stress (Martin et al., 2001). Moreover, fewer social and individual resources to cope with life stressors are available to individuals as they grow older (Martin et al., 2001). Aside from depleting resources, older individuals’ sense of perceived personal control may decline, affecting their response to stressors (Cairney and Krause, 2008). Overall, older individuals may have a reduced sense of control regarding their life events, bodies, and health, which exacerbates stressors associated with aging.

The results also indicate that increased feelings of security may protect from depression and stress among the participants. Based on the CDSQ questions, security relates to feelings of peace and a friendly and loving world. This finding aligns with previous studies, which observed peace of mind lessens depression and stress (Liang et al., 2018). Furthermore, the protective effects of such secure feelings about oneself and the world could stem from the protective effect conferred by confidence in a robust social support system that may be leveraged during hardships (Serrano et al., 2023).

Overall, the present study’s results support its hypothesis that spirituality significantly influences mental health. The dimension of spirituality, particularly belief in God, can be a risk factor for depression. Moreover, feelings of security may act as protective buffers against depression and stress. Contrastingly, the results support the alternative hypothesis that sociodemographic characteristics have a statistically significant influence on mental health, where being LGBTQ+ may be a risk factor for depression, anxiety, and stress. Likewise, older age may be a risk factor for stress.

### 4.1 Limitations

To our knowledge, this is the first study that explored the relationship between sociodemographic characteristics, spirituality, and mental health among emerging adults in Metro Manila. Nonetheless, its findings must be considered with various limitations. First, it utilized a cross-sectional design, which cannot determine the causality between the studied variables. Future studies may use prospective designs to assess the causal relationship between the variables. Second, the study utilized a non-probability sampling design. Therefore, the present study may not adequately represent emerging adults from Metro Manila. Third, the study was conducted in a single urbanized region in the Philippines, thus limiting its findings to that of Metro Manila. The participants were predominantly female, middle to upper class, and were limited to emerging adults. Future studies can be conducted on older individuals and explore the effects of religiosity on the mental health of LGBTQ+ persons. Moreover, to improve the study design, future studies could utilize a representative sample and probability sampling, examine the studied variables as possible moderators using Hayes’s (2018) PROCESS macro in SPSS, broaden the study setting, consider other factors such as political orientation, and ensure the adequate representation of males, lower-income classes, and older emerging adults.

## 5 Conclusion

Depression was higher than anxiety and stress among the participants. Being LGBTQ+ also increases the risk of depression, anxiety, and stress. Likewise, belief in God and older age act as risk factors for depression and stress, respectively. Contrastingly, feelings of security may be protective against depression and stress. Similarly, being LGBTQ+ may increase the risk for depression, anxiety, and stress due to their higher experiences of discrimination, stigma, and lack of family acceptance. Belief in God could also be a risk factor for depression, which could be further explored in subsequent studies. Moreover, older age could be a risk factor for stress due to the loss of control experienced by aging individuals regarding their health, body, and life experiences. Lastly, a greater sense of security about the self and the world could stem from their confidence in their social support system.

### 5.1 Practice implications

LGBTQ+ emerging adults and those with older age may need additional focus in mental health programs. Developing mental health programs may help enhance a person’s individual security and be effective in the Philippine context. During the aftermath of Typhoon Haiyan in 2013, the Psychological Association of the Philippines (PAP) developed interventions to respond to the needs of disaster survivors. The resilience program, named *Katatagan*, was shown to increase its recipients’ self-efficacy, managing physical reactions, emotions, cognition, problem-solving, social support, and giving hope (Hechanova, 2019). Despite these interventions being effective in natural calamities and given the study’s limitations, further research is needed to understand the relationship between mental health, sociodemographic characteristics, and spirituality among emerging Filipino adults.

## Data Availability

The original contributions presented in the study are included in the article/supplementary material, further inquiries can be directed to the corresponding author.

## References

[ref1] Akré E. R. Anderson A. Stojanovski K. Chung K. W. VanKim N. A. Chae D. H. (2021). Depression, anxiety, and alcohol use among LGBTQ+ people during the COVID-19 pandemic. Am. J. Public Health 111, 1610–1619. doi: 10.2105/ajph.2021.306394, PMID: 34410817 PMC8589058

[ref2] Alibudbud R. (2023a). Gender in climate change: safeguarding LGBTQ+ mental Health in the Philippine climate change response from a minority stress perspective. J. Prev. Med. Public Health 56, 196–199. doi: 10.3961/jpmph.22.501, PMID: 37055362 PMC10111101

[ref3] Alibudbud R. (2023b). Gender in mental health: relationship of spirituality, social support, and COVID-19-related fear among heterosexual and LGBTQ+ youth. Front. Sociol. 7:1102664. doi: 10.3389/fsoc.2022.1102664, PMID: 36687008 PMC9846850

[ref4] Alibudbud R. (2024). Navigating the Philippine mental health system for the nation’s youth: challenges and opportunities. BJPsych Int. 21, 56–58. doi: 10.1192/bji.2024.5 PMC12022871 40291986

[ref5] Benatov J. Ochnik D. Rogowska A. M. Arzenšek A. Bitenc U. M. (2022). Prevalence and sociodemographic predictors of mental Health in a representative sample of young adults from Germany, Israel, Poland, and Slovenia: a longitudinal study during the COVID-19 pandemic. Int. J. Environ. Res. Public Health 19:1334. doi: 10.3390/ijerph19031334, PMID: 35162364 PMC8835083

[ref6] Bonelli R. Dew R. E. Koenig H. G. Rosmarin D. H. Vasegh S. (2012). Religious and spiritual factors in depression: review and integration of the research. Depress. Res. Treat. 2012:962860. doi: 10.1155/2012/962860, PMID: 22928096 PMC3426191

[ref7] Cacho R. del Castillo F. (2022). God’s benevolent love in the time of COVID-19 pandemic: articulations and experiences of select Filipino youth. Religions 13:162. doi: 10.3390/rel13020162

[ref8] Cairney J. Krause N. (2008). Negative life events and age-related decline in mastery: are older adults more vulnerable to the control-eroding effect of stress? J. Gerontol. Ser. B Psychol. Sci. Soc. Sci. 63, S162–S170. doi: 10.1093/geronb/63.3.S162 18559691

[ref9] Charzyńska E. Heszen-Celińska I. (2019). Spirituality and mental health care in a religiously homogeneous country: definitions, opinions, and practices among polish mental health professionals. J. Relig. Health 59, 113–134. doi: 10.1007/s10943-019-00911-w, PMID: 31512031 PMC6976552

[ref10] Cleofas J. V. Alibudbud R. C. (2023). Emerging from a two-year-long quarantine: a retrospective study on life satisfaction trajectory and depression among young LGBTQ+ students in the Philippines. SAGE Open Nurs. 9:23779608231158980. doi: 10.1177/23779608231158980, PMID: 36861049 PMC9969438

[ref11] Cohen J. (1988). Statistical power analysis for the behavioral sciences. New York, NY: Routledge Academic.

[ref12] Cureg E. Sawali R. Aquino M. (2023). Underscoring the Mental Health Agenda in the Philippines. Philippines: Socioeconomic Research Portal for the Philippines.

[ref13] Ede S. S. Ugwuodo E. P. Okoh C. F. Egbumike C. J. Chukwu D. A. Irem F. O.. (2021). Impact of religious participation and spirituality on the Health of Nigerian older people: an online survey. J. Relig. Spiritual. Aging 35, 56–70. doi: 10.1080/15528030.2021.2001407

[ref14] Faul F. Erdfelder E. Lang A. Buchner A. (2007). G*power 3: a flexible statistical power analysis program for the social, behavioral, and biomedical sciences. Behav. Res. Methods 39, 175–191. doi: 10.3758/bf03193146, PMID: 17695343

[ref15] Ganson K. T. Tsai A. C. Weiser S. D. Benabou S. E. Nagata J. M. (2021). Job insecurity and symptoms of anxiety and depression among US young adults during COVID-19. J. Adolesc. Health 68, 53–56. doi: 10.1016/j.jadohealth.2020.10.008, PMID: 33183926

[ref16] Gruber J. Prinstein M. J. Clark L. A. Rottenberg J. Abramowitz J. S. Albano A. M.. (2020). Mental health and clinical psychological science in the time of COVID-19: challenges, opportunities, and a call to action. Am. Psychol. 76, 409–426. doi: 10.1037/amp0000707, PMID: 32772538 PMC7873160

[ref17] Harder B. M. Sumerau J. E. (2018). Understanding gender as a fundamental cause of Health: simultaneous linear relationships between gender, mental Health, and physical Health over time. Sociol. Spectr. 38, 387–405. doi: 10.1080/02732173.2018.1532366

[ref18] Hardt J. Schultz S. Xander C. Becker G. Dragan M. (2012). The spirituality questionnaire: Core dimensions of spirituality. Psychology 3, 116–122. doi: 10.4236/psych.2012.31017

[ref19] Hasin D. S. Sarvet A. L. Meyers J. L. Saha T. D. Ruan W. J. Stohl M.. (2018). Epidemiology of adult DSM-5 major depressive disorder and its specifiers in the United States. JAMA Psychiatry 75, 336–346. doi: 10.1001/jamapsychiatry.2017.4602, PMID: 29450462 PMC5875313

[ref20] Hawes M. Szenczy A. K. Klein D. N. Hajcak G. Nelson B. D. (2021). Increases in depression and anxiety symptoms in adolescents and young adults during the COVID-19 pandemic. Psychol. Med. 52, 3222–3230. doi: 10.1017/s0033291720005358, PMID: 33436120 PMC7844180

[ref21] Hayes A. F. (2018). Introduction to mediation, moderation, and conditional Process analysis: a regression-based approach (methodology in the social sciences). New York, NY: The Guilford Press.

[ref22] Hechanova M. R. M. (2019). Development of community-based mental health interventions in the Philippines: an ecological perspective. Psychol. Res. Urban Soc. 2, 10–25. doi: 10.7454/proust.v2i1.41

[ref23] Heise L. Greene M. B. Opper N. Stavropoulou M. Harper C. Nascimento M.. (2019). Gender inequality and restrictive gender norms: framing the challenges to health. Lancet 393, 2440–2454. doi: 10.1016/s0140-6736(19)30652-x 31155275

[ref24] Hunter J. Butler C. Cooper K. (2021). Gender minority stress in trans and gender diverse adolescents and young people. Clin. Child Psychol. Psychiatry 26, 1182–1195. doi: 10.1177/13591045211033187, PMID: 34293962

[ref25] Inglis G. Jenkins P. McHardy F. Sosu E. Wilson C. (2022). Poverty stigma, mental health, and well-being: a rapid review and synthesis of quantitative and qualitative research. J. Community Appl. Soc. Psychol. 33, 783–806. doi: 10.1002/casp.2677

[ref26] Jeppsen B. Winkeljohn Black S. Pössel P. Rosmarin D. H. (2022). Does closeness to god mediate the relationship between prayer and mental health in Christian, Jewish, and Muslim samples? Ment. Health Relig. Cult. 25, 99–112. doi: 10.1080/13674676.2021.2024801

[ref27] Kao L. E. Peteet J. R. Cook C. C. H. (2020). Spirituality and mental health. J. Study Spiritual. 10, 42–54. doi: 10.1080/20440243.2020.1726048

[ref28] Kibrik E. L. Cohen N. Stolowicz-Melman D. Levy A. Boruchovitz-Zamir R. Diamond G. M. (2018). Measuring adult children’s perceptions of their parents’ acceptance and rejection of their sexual orientation: initial development of the parental acceptance and rejection of sexual orientation scale (PARSOS). J. Homosex. 66, 1513–1534. doi: 10.1080/00918369.2018.1503460, PMID: 30142289

[ref29] Kraemer H. C. Blasey C. M. (2004). Centring in regression analyses: a strategy to prevent errors in statistical inference. Int. J. Methods Psychiatr. Res. 13, 141–151. doi: 10.1002/mpr.170, PMID: 15297898 PMC6878533

[ref30] Lakens D. (2013). Calculating and reporting effect sizes to facilitate cumulative science: a practical primer for t-tests and ANOVAs. Front. Psychol. 4:863. doi: 10.3389/fpsyg.2013.00863, PMID: 24324449 PMC3840331

[ref31] Lawrence R. E. Brent D. Mann J. J. Burke A. K. Grunebaum M. F. Galfalvy H. C.. (2016). Religion as a risk factor for suicide attempt and suicide ideation among depressed patients. J. Nerv. Ment. Dis. 204, 845–850. doi: 10.1097/NMD.0000000000000484, PMID: 26894320 PMC4990512

[ref32] Lee C. M. Cadigan J. M. Rhew I. C. (2020). Increases in loneliness among young adults during the COVID-19 pandemic and association with increases in mental health problems. J. Adolesc. Health 67, 714–717. doi: 10.1016/j.jadohealth.2020.08.009, PMID: 33099414 PMC7576375

[ref33] Lê-Scherban F. Brenner A. B. Schoeni R. F. (2016). Childhood family wealth and mental health in a national cohort of young adults. SSM Popul. Health 2, 798–806. doi: 10.1016/j.ssmph.2016.10.008, PMID: 28584861 PMC5455782

[ref34] Leung C. H. Mu Y. (2021). Spiritual and mental health of teenagers in Hong Kong and in mainland China under the impact of COVID-19. Asian Educ. Dev. Stud. 11, 340–355. doi: 10.1108/aeds-04-2021-0076

[ref35] Liang H. Chen C. Li F. Wu S. Wang L. Zheng X.. (2018). Mediating effects of peace of mind and rumination on the relationship between gratitude and depression among Chinese university students. Curr. Psychol. 39, 1430–1437. doi: 10.1007/s12144-018-9847-1

[ref36] Libiran T. J. D. Cepeda R. L. C. Ramos C. K. M. Carlo J. Alano O. Guballa M. J. S. (2024). Understanding the challenges faced by Filipino LGBTQ+ individuals with strong religious ties. Int. J. Res. Innov. Soc. Sci. 8, 2520–2547. doi: 10.47772/IJRISS.2024.801186

[ref37] Ljungqvist I. Topor A. Forssell H. Svensson I. Davidson L. (2015). Money and mental illness: a study of the relationship between poverty and serious psychological problems. Community Ment. Health J. 52, 842–850. doi: 10.1007/s10597-015-9950-9, PMID: 26433374

[ref38] Lovibond S. H. Lovibond P. F. (1995). Manual for the Depression Anxiety Stress Scale. Sydney: The Psychological Foundation of Australia.

[ref40] Maravilla N. M. A. T. Tan M. J. T. (2021). Philippine mental Health act: just an act? A call to look into the bi-directionality of mental health and economy. Front. Psychol. 12:706483. doi: 10.3389/fpsyg.2021.706483, PMID: 34367032 PMC8334355

[ref41] Martin M. Grünendahl M. Martin P. (2001). Age differences in stress, social resources, and well-being in middle and older age. J. Gerontol. Ser. B Psychol. Sci. Soc. Sci. 56, P214–P222. doi: 10.1093/geronb/56.4.P214 11445607

[ref42] Martinez A. B. Co M. Lau J. Brown J. S. (2020). Filipino help-seeking for mental health problems and associated barriers and facilitators: a systematic review. Soc. Psychiatry Psychiatr. Epidemiol. 55, 1397–1413. doi: 10.1007/s00127-020-01937-2, PMID: 32816062 PMC7578164

[ref43] Mental Health Facilities (2022. Free mental health services (UPDATED 2022 LIST). Available at: https://www.resilientlgus.ph/content/free-mental-health-services-updated-2022-list (Accessed November 21, 2023).

[ref44] Mental Health PH. (2016). Directory: Mental Health PH. Available at: https://mentalhealthph.org/directory/ (Accessed November 21, 2023).

[ref45] Meyer I. H. (2003). Prejudice, social stress, and mental health in lesbian, gay, and bisexual populations: conceptual issues and research evidence. Psychol. Bull. 129, 674–697. doi: 10.1037/0033-2909.129.5.674, PMID: 12956539 PMC2072932

[ref47] Palaniswamy U. Ponnuswami I. (2012). Spirituality and mental Health among the elderly practising spirituality. J. Res. Exten. Develop. 1, 8–13. Available at: https://www.researchgate.net/publication/236325849_Spirituality_and_Mental_Health_among_the_Elderly_practising_Spirituality

[ref48] Piccinini C. R. P. De Castro Almeida V. Da Silva Ezequiel O. De Matos Fajardo E. F. Lucchetti A. L. G. Lucchetti G. (2021). Religiosity/spirituality and mental health and quality of life of early pregnant women. J. Relig. Health 60, 1908–1923. doi: 10.1007/s10943-020-01124-2, PMID: 33386569

[ref49] Pieh C. Budimir S. Probst T. (2020). The effect of age, gender, income, work, and physical activity on mental health during coronavirus disease (COVID-19) lockdown in Austria. J. Psychosom. Res. 136:110186. doi: 10.1016/j.jpsychores.2020.110186, PMID: 32682159 PMC7832650

[ref51] Ridley M. Rao G. Schilbach F. Patel V. (2020). Poverty, depression, and anxiety: causal evidence and mechanisms. Science 370:eaay0214. doi: 10.1126/science.aay0214, PMID: 33303583

[ref52] Rosmarin D. H. Green D. Pirutinsky S. McKay D. (2013). Attitudes toward spirituality/religion among members of the Association for Behavioral and Cognitive Therapies. Prof. Psychol. Res. Pract. 44, 424–433. doi: 10.1037/a0035218

[ref53] Ryan C. Russell S. T. Huebner D. Diaz R. Sanchez J. (2010). Family acceptance in adolescence and the health of LGBT young adults. J. Child Adolesc. Psychiatr. Nurs. 23, 205–213. doi: 10.1111/j.1744-6171.2010.00246.x, PMID: 21073595

[ref55] Serrano I. M. A. Cuyugan A. M. N. Cruz K. Mahusay J. M. A. Alibudbud R. (2023). Sociodemographic characteristics, social support, and family history as factors of depression, anxiety, and stress among young adult senior high school students in metro Manila, Philippines, during the COVID-19 pandemic. Front. Psych. 14:1225035. doi: 10.3389/fpsyt.2023.1225035, PMID: 37772068 PMC10525313

[ref56] Sharma V. Marin D. B. Koenig H. Feder A. Iacoviello B. M. Southwick S. M.. (2017). Religion, spirituality, and mental health of U.S. military veterans: results from the National Health and resilience in veterans study. J. Affect. Disord. 217, 197–204. doi: 10.1016/j.jad.2017.03.071, PMID: 28415007

[ref57] Soled K. R. S. Clark K. D. Altman M. R. Bosse J. D. Thompson R. A. Squires A.. (2022). Changing language, changes lives: learning the lexicon of LGBTQ+ health equity. Res. Nurs. Health 45, 621–632. doi: 10.1002/nur.22274, PMID: 36321331 PMC9704510

[ref58] Solomou I. Constantinidou F. (2020). Prevalence and predictors of anxiety and depression symptoms during the COVID-19 pandemic and compliance with precautionary measures: age and sex matter. Int. J. Environ. Res. Public Health 17:4924. doi: 10.3390/ijerph17144924, PMID: 32650522 PMC7400373

[ref59] Ta-asan K. (2022). Who are the middle class in the Philippines? Philippine institute for developmental studies. Available at: https://pidswebs.pids.gov.ph/CDN/document/1653265260_628ad36c07ed2.pdf (Accessed October 15, 2023).

[ref60] Tan K. K. H. Saw A. T. W. (2022). Prevalence and correlates of mental health difficulties amongst LGBTQ people in Southeast Asia: a systematic review. J. Gay Lesbian Ment. Health 27, 401–420. doi: 10.1080/19359705.2022.2089427

[ref61] Thomas J. Barbato M. (2020). Positive religious coping and mental Health among Christians and Muslims in response to the COVID-19 pandemic. Religions 11:498. doi: 10.3390/rel11100498

[ref62] Vaingankar J. A. Choudhary N. Chong S. A. Kumar F. D. S. Abdin E. Shafie S.. (2021). Religious affiliation in relation to positive mental health and mental disorders in a multi-ethnic asian population. Int. J. Environ. Res. Public Health 18:3368. doi: 10.3390/ijerph18073368, PMID: 33805121 PMC8038033

[ref63] Valdiserri R. O. Holtgrave D. R. Poteat T. C. Beyrer C. (2019). Unraveling health disparities among sexual and gender minorities: a commentary on the persistent impact of stigma. J. Homosex. 66, 571–589. doi: 10.1080/00918369.2017.1422944, PMID: 29297774

[ref64] Walters S. J. Campbell M. J. (2004). The use of bootstrap methods for analysing Health-related quality of life outcomes (particularly the SF-36). Health Qual. Life Outcomes 2:70. doi: 10.1186/1477-7525-2-70, PMID: 15588308 PMC543443

